# Collagen matrix vs mitomycin-C in trabeculectomy and combined phacoemulsification and trabeculectomy: a randomized controlled trial

**DOI:** 10.1186/s12886-016-0393-z

**Published:** 2016-12-29

**Authors:** Angelo P. Tanna, Alfred W. Rademaker, C. Gustavo de Moraes, David G. Godfrey, Steven R. Sarkisian, Steven D. Vold, Robert Ritch

**Affiliations:** 1 Department of Ophthalmology, Northwestern University Feinberg School of Medicine, 645 N. Michigan Ave., Suite 440, Chicago, IL 60611 USA; 2 Department of Preventive Medicine, Northwestern University Feinberg School of Medicine, Chicago, IL USA; 3 Edward S. Harkness Eye Institute, Columbia University Medical Center, New York, USA; 4 Glaucoma Associates of Texas, Dallas, TX USA; 5 Dean McGee Eye Institute, Oklahoma City, OK USA; 6 Vold Vision, Fayetteville, AR USA; 7 Einhorn Clinical Research Center, New York Eye and Ear Infirmary of Mount Sinai, New York, NY USA

## Abstract

**Background:**

Antifibrotic agents are commonly utilized to enhance the success rates of trabeculectomy. Novel approaches to further improve success rates and reduce the risks of complications are needed. The purpose of this study was to compare intraocular pressure (IOP)-lowering efficacy and safety of trabeculectomy or combined phacoemulsification and trabeculectomy with mitomycin-C (MMC) vs. Collagen Matrix (CM).

**Methods:**

A prospective, multicenter, randomized controlled trial was performed. Ninety-five eyes of 94 patients with uncontrolled glaucoma despite medical therapy, without previous incisional glaucoma surgery underwent trabeculectomy (85 eyes) or combined phacoemulsification and trabeculectomy (10 eyes) and were randomized to MMC or CM. One eye of each subject was analyzed. Patients were followed for 24 months. The criteria for complete success were IOP >5 and ≤21 mmHg with at least a 20% reduction below medicated baseline without additional glaucoma surgery or medications. The main outcome measures were complete success rates at 24 months with Kaplan-Meier analysis and incidence of adverse events.

**Results:**

The baseline IOPs were 20.4 ± 6.0 mmHg and 21.2 ± 6.1 (mean ± standard deviation, *p* = 0.49) on 3.2 ± 1.1 and 3.1 ± 1.0 medications (*p* = 0.53) compared to 11.8 ± 5.2 and 12.8 ± 3.7 (*p* = 0.36) on 0.5 ± 0.8 and 0.6 ± 1.0 medications (*p* = 0.63) at 2 years in the MMC and CM groups, respectively. Kaplan-Meier analysis demonstrated complete success rates were similar in both groups at 24 months: 38.4 ± 7.6% with MMC and 56.2 ± 7.9% with CM (mean ± standard error, *p* = 0.112, log rank test); however, a significantly higher incidence of failure due to persistent hypotony was observed with MMC (*p* = 0.002).

**Conclusions:**

Use of the CM implant at the time of trabeculectomy or combined phacoemulsification and trabeculectomy is associated with similar complete success rates compared to adjunctive MMC; however, the risk of persistent hypotony is higher with MMC.

**Trial registration:**

ClinicalTrials.gov registration number NCT01440751.

Registered 9/14/11

**Electronic supplementary material:**

The online version of this article (doi:10.1186/s12886-016-0393-z) contains supplementary material, which is available to authorized users.

## Background

Trabeculectomy remains the most frequently utilized operation for eyes with severe glaucoma in which a low target intraocular pressure (IOP) is desired [1]. Antifibrotic therapy with mitomycin-C (MMC) or 5-fluorouracil (5-FU) is a valuable adjunct to trabeculectomy for reducing scarring and improving IOP-lowering efficacy. Although extremely variable, due in part to different definitions of success, demographic characteristics of subjects and duration of follow-up, some recently reported success and complication rates are less than desirable [2–6].

Hsu et al. [7] demonstrated in a rabbit model of conjunctival wound healing that full-thickness conjunctival defects healed with less wound contraction and the formation of more normal-appearing conjunctival stroma when the wounds were grafted with a porous collagen matrix composed of a collagen-glycosaminoglycan copolymer compared to control eyes in which the conjunctival defects were left bare. Chen et al. [8] further advanced the concept of using a porous collagen matrix (CM) implant as an adjunct to trabeculectomy. Their approach was to use the implant to resist over-filtration in the early post-operative period by acting as a physical barrier and to maintain long-term pressure control by promoting the development of a loosely organized scar as the implant degrades. In a rabbit model, eyes undergoing trabeculectomy with CM implantation maintained IOP reduction to 55% below baseline at day 28, whereas control eyes undergoing trabeculectomy without CM reverted to baseline IOP levels by day 21 [8].

The Ologen CM (Aeon Astron Europe B.V., Leiden, The Netherlands) is a porous, disc-shaped implant that is commercially available in two sizes, a 6-mm diameter disc with a thickness of 2 mm and a 12-mm diameter disc with a thickness of 1 mm. It is composed of a collagen-glycosaminoglycan copolymer and was developed for use as an adjunctive device for trabeculectomy surgery. The CM serves as a spacer and a scaffolding to modulate the fibrotic response as fibroblasts and myofibroblasts proliferate in response to surgically induced tissue injury. We hypothesized adjunctive use of the CM at the time of trabeculectomy would result in similar IOP outcomes but fewer adverse events compared to MMC.

The success of the use of trabeculectomy with CM compared to adjunctive therapy with MMC has varied with respect to the reported outcomes. In a recently published meta-analysis, the authors found that outcomes were similar, but pointed out that additional randomized clinical trials are needed [9]. We report herein the results of a multicenter randomized clinical trial designed to compare efficacy and safety of trabeculectomy or combined phacoemulsification and trabeculectomy with MMC versus CM.

## Methods

This study was approved by five Institutional Review Boards with oversight authority of the eight study sites. The study followed the tenets of the Declaration of Helsinki, is in compliance with the Health Insurance Portability and Accountability Act of 1996 and is registered with ClinicalTrials.gov (NCT01440751). An independent data safety monitoring committee received regular reports on surgical outcomes and adverse events during the course of the study and at its conclusion. The coordinating center at New York Eye and Ear Infirmary collected all data regarding the study using a web-based reporting system.

### Inclusion/exclusion criteria

Patients were enrolled at 8 clinical centers, including private practices and academic medical centers between February 2012 and December 2012. All clinical investigators were glaucoma subspecialists. Subjects, who were 30 years–of-age or older and in whom trabeculectomy or combined phacoemulsification and trabeculectomy had been recommended to manage glaucoma that was uncontrolled despite medical therapy, were enrolled. The rationale behind the decision to perform incisional glaucoma surgery was at the discretion of the individual surgeons. Patients were eligible if they had primary open-angle glaucoma, exfoliation glaucoma, pigmentary glaucoma, corticosteroid-induced glaucoma, or primary angle-closure glaucoma. Glaucoma was defined as the presence of optic disc excavation associated with a visual field defect on standard automated perimetry. Patients with neovascular glaucoma, glaucoma associated with uveitis, or glaucoma in aphakic eyes were excluded, as were those with prior ocular surgery involving a conjunctival incision. Pseudophakic patients who had undergone cataract surgery with a clear corneal incision were eligible for participation.

All subjects underwent a comprehensive ophthalmological examination. Baseline IOP was the mean of 2 measurements on 2 separate study visits within 90 days of surgery. IOP measurements throughout the study were obtained by an examiner and a separate reader using a calibrated Goldmann applanation tonometer. Two measurements were obtained for the study eye and the mean was used. If the difference between readings was greater than 2 mmHg, an additional reading was obtained and the median measurement was used.

Postoperative study visits occurred on postoperative days 1, 7, 14, 30, and 90 and months 6, 12, 18, and 24. Automated static perimetry with the Humphrey Visual Field 24–2 algorithm was performed at baseline, at months 6, 12, 18, and 24. Stereoscopic optic disc photographs were obtained at baseline and at months 12 and 24. Refractions were performed and best spectacle corrected visual acuity was measured at each study visit.

The randomization process used a sealed envelope system, the treatment assignment being revealed at the time of surgery. Subjects were randomized to trabeculectomy with MMC or trabeculectomy with the Ologen CM. A 1:1 randomization scheme to the two study arms in blocks of 15, by study site, was used to ensure that each site had a similar number of subjects assigned to the each of the two groups.

### Surgical procedure

The trabeculectomy was performed according to surgeon preference. For the MMC group, the concentration of the drug was 0.4 mg/mL. The duration of application was at the discretion of the surgeon. Manipulation of scleral flap sutures with laser suture lysis, removal of releasable sutures and trans-conjunctival needle revision (TCNR) were performed at the discretion of the surgeon and were not counted as complications or as indicators of surgical failure.

For the CM group, no intraoperative antifibrotic therapy was utilized. The CM used was the 12-mm diameter disc with a thickness of 1 mm. The disc was placed over the scleral flap and under the conjunctiva prior to closure.

### Complete and qualified success criteria

The criteria for the definition of complete success were IOP >5 and ≤21 mmHg with at least a 20% reduction below the mean of the two baseline study visit IOPs, and without subsequent ocular hypotensive medication or additional glaucoma surgery. Qualified success was defined by the same criteria but with one or more ocular hypotensive medications to meet IOP criteria. Overall success was the combined complete and qualified success rates. Failure was defined if any of the IOP criteria for success were unmet on two consecutive study visits starting with the six-month visit; however, the date of failure was defined as the *first* study visit when at least one of the criteria was unmet. If failure based on IOP criteria occurred at the final study visit (at the 24 month visit or an earlier visit in subjects for whom additional follow-up is unavailable), failure was defined to have occurred on that final visit even if failure criteria were not met on two consecutive visits.

An alternate definition of success with a more stringent upper limit threshold of ≤17 mmHg (a commonly used cutpoint in glaucoma clinical trials) was also evaluated. For both sets of criteria, ≤17 and ≤21 mmHg, qualified success was defined to have occurred if one or more ocular hypotensive medications was in use. Beginning with the six-month visit, as soon as an ocular hypotensive medication was in use at the time of a study visit, only qualified success could be achieved, even if medications were later withdrawn and success criteria were met.

If additional glaucoma surgery was performed, failure was defined to have occurred on that date, at any point in the study, even prior to the six-month visit. Needle revisions were not classified as additional glaucoma surgery.

For each of the four Kaplan-Meier survival curves generated with the above criteria for success and overall success, log-rank tests were used to test for statistical significance between the two groups. As an ancillary analysis, the same procedures were repeated after excluding the subjects who underwent combined phacoemulsification surgery and trabeculectomy. Similarly, the data were also reanalyzed after reclassifying as successes the subjects who failed by meeting the numerical definition of hypotony (≤5 mmHg), but in whom there were no structural (hypotony maculopathy, optic disc edema, choroidal detachment) or functional (loss of visual acuity) consequences of the low IOP and in whom no surgical intervention was utilized to correct the hypotony.

### Ocular hypotensive medical therapy

A washout was not required at the time of enrollment or for the baseline study visits. Throughout the clinical trial, the use of ocular hypotensive medications was at the discretion of the each investigator. The number of medications in use was determined based on the number of ocular hypotensive medication classes used. A fixed combination agent with two classes of medication was counted as two medications. The mean number of medications used in each group, at each study visit, was compared with the Mann–Whitney *U* test.

### Adverse events

Throughout the study, all adverse events were recorded and submitted to the coordinating center. Hypotony-related complications were recorded at each study visit.

Hypotony was defined as IOP ≤5 mmHg on two consecutive visits occurring at the six month visit or thereafter. Additionally, hypotony was defined to have occurred if the IOP was ≤5 mmHg at the final visit. A Kaplan-Meier analysis with a log-rank test for significance was performed to ascertain differences in the incidence of hypotony between groups. An alternate statistical analysis was performed after excluding the cases of hypotony in which, although the IOP was ≤5 mmHg, there were no structural or functional consequences of the low IOP as described above.

### Power and sample size calculation

The original power calculation determined that 64 patients per arm were needed to detect a late hypotony rate of 24.8% in the MMC group versus 5.0% in the CM group, with 80% power with a two-tailed test and a Type I error rate of 5%. Power was maintained at 80% to detect the observed late hypotony rates of 18.8% in the MMC group of 48 patients versus 0% in the CM group of 46 patients (1% rate assumed for power calculation) with a two-tailed .05 level test.

A non-inferiority power calculation for the hazard ratio (failure rate in CM vs failure rate in MMC) indicated that with the study sample size, there was 80% power to detect a non-inferiority margin up to 1.87 with a 5% Type I error rate. A proportional hazards analysis of complete success gives a hazard ratio of 0.65 with an upper confidence limit of 1.08. This indicates that the failure rate in the CM group is well below that in the MMC group with a worst case scenario being only 8% higher than the MMC group.

### Statistical analysis

For continuous measures such as IOP, IOP reduction, number of medications and visual acuity, group comparisons were made at each time point with an independent sample *t*-test. Results were verified with the Wilcoxon rank sum test. Categorical variables were compared between groups with Fisher’s exact test. Time to success was summarized with Kaplan-Meier curves and comparisons made with the log-rank test. Success rates were compared between groups adjusting for age, sex and combined subsequent or prior cataract surgery with Cox proportional hazards regression. An intent-to-treat analysis was performed with two exceptions. One patient in the MMC group did not undergo trabeculectomy, was withdrawn from the study at the time of surgery and did not have any follow-up and outcome data. Both eyes of one patient were randomized; one eye was randomly selected for statistical analysis. Alpha-level was set at 5%.

## Results

**Fig. 1:**
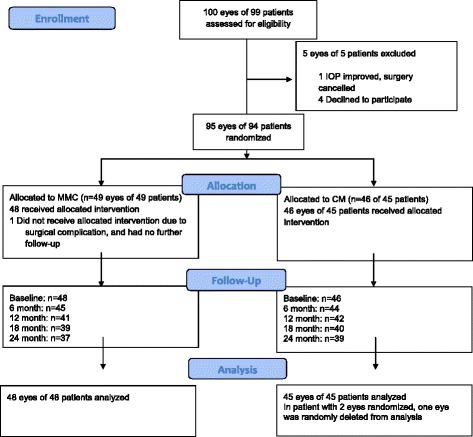
Patient flow chart from enrollment through 24 months. Abbreviations: IOP = Intraocular Pressure; MMC = Mitomycin C; CM = Collagen Matrix

One hundred potential eyes of 99 subjects were assessed for eligibility (Fig. 1). Four declined to participate and a decision was made to cancel the plan for trabeculectomy in one subject due to improved IOP. Ninety-five eyes of 94 subjects were randomized. One subject allocated to the MMC group did not undergo trabeculectomy due to an intraoperative cataract surgery complication – this subject was not included in the analysis because no post-operative data were available. In a protocol violation, both eyes of one subject were enrolled. Both eyes were randomized to the CM group. One eye was randomly selected for analysis. Results are reported for 48 eyes of 48 patients in the MMC group and 45 eyes of 45 patients in the CM group. Demographic and baseline clinical characteristics were statistically similar between groups and are summarized in Table 1.

**Table 1:** Demographics and baseline characteristics

		Mitomycin-C (*n* = 48)	Collagen matrix (*n* = 45)	*p*-Value (Fisher’s Exact Test)
Sex	N (% Male)	28 (58%)	21 (47%)	0.18
Age (Years)	Mean (SD)	71.1 (10.0)	69.4 (10.7)	0.41
	Median (IQR)	71.5 (65–79)	71.0 (63–79)	
Race	European-Derived	38 (79%)	33 (73%)	0.42 (one *p*-value for multicategory race)
	African-Derived	8 (17%)	9 (20%)	
	Asian	1 (2%)	2 (4%)	
	Other	1 (2%)	1 (2%)	
Diagnosis N (%)	Primary Open Angle Glaucoma	43	38	0.35 (one *p*-value for multicategory diagnosis)
	Pigmentary Glaucoma	0	3	
	Primary Angle Closure Glaucoma	3	2	
	Corticosteroid Induced Glaucoma	1	0	
	Pseudoexfoliation Glaucoma	1	1	
	Mixed Mechanism	0	1	
Visual Acuity (Log MAR)	Mean (SD)	0.18 (0.26)	0.17 (0.23)	0.81
	Median (IQR)	0.10 (0–0.21)	0.10 (0–0.30)	
Visual Field Mean Deviation	Mean (SD)	−14.1 (10.7)	−12.9 (8.6)	0.55
	Median (IQR)	−14.4 (−23.3 − −6.4)	−14.1 (−19.9 − −5.8)	
Baseline IOP (mm Hg)	Mean (SD)	20.4 (6.0)	21.2 (6.1)	0.49
	Median (IQR)	19.8 (16.0–23.6)	19.0 (17.0–26.0)	
Number of Medications	Mean (SD)	3.21 (1.13)	3.07 (1.00)	0.53
	Median (IQR)	3 (2–4)	3 (2–4)	0.48

*SD* standard deviation, *IQR* interquartile range, *Log MAR* logarithm of the minimum angle of resolution, *IOP* intraocular pressure

Data regarding cataract surgery are summarized in Table 2. Despite randomization, 22 subjects in the MMC group and 11 in the CM group were pseudophakic at the time of enrollment (*p* = 0.05). Four subjects in the MMC group and 6 subjects in the CM group underwent combined cataract surgery, intraocular lens implantation and trabeculectomy (combined surgery, *p* = 0.51). Due to the small number of combined surgery cases, a statistical analysis of success in that subgroup would not be meaningful and was not performed. During the two-year follow-up interval, 7 eyes in the MMC group and 6 in the CM group underwent cataract surgery. The proportions of eyes in the two groups that underwent cataract surgery during the follow-up interval, expressed as a proportion of those at risk (i.e., those that were phakic) were statistically similar between groups (*p* = 0.32).

**Table 2:** Cataract surgery

		Mitomycin-C (*n* = 48)	Collagen Matrix (*n* = 45)	*p*-value (Fisher's Exact Test)
Phacoemulsification Prior to Enrollment		22 (45.8%)	11 (24.4%)	0.05
Combined Phacoemulsification/ Trabeculectomy		4 (8.3%)	6 (13.3%)	0.51
Phacoemulsification After Trabeculectomy, During Study Period		7	5	
	Number at Risk	22	28	
	Rate	31.8%	17.9%	0.32

### Intraocular pressure, visual acuity and visual field outcomes

**Fig. 2:**
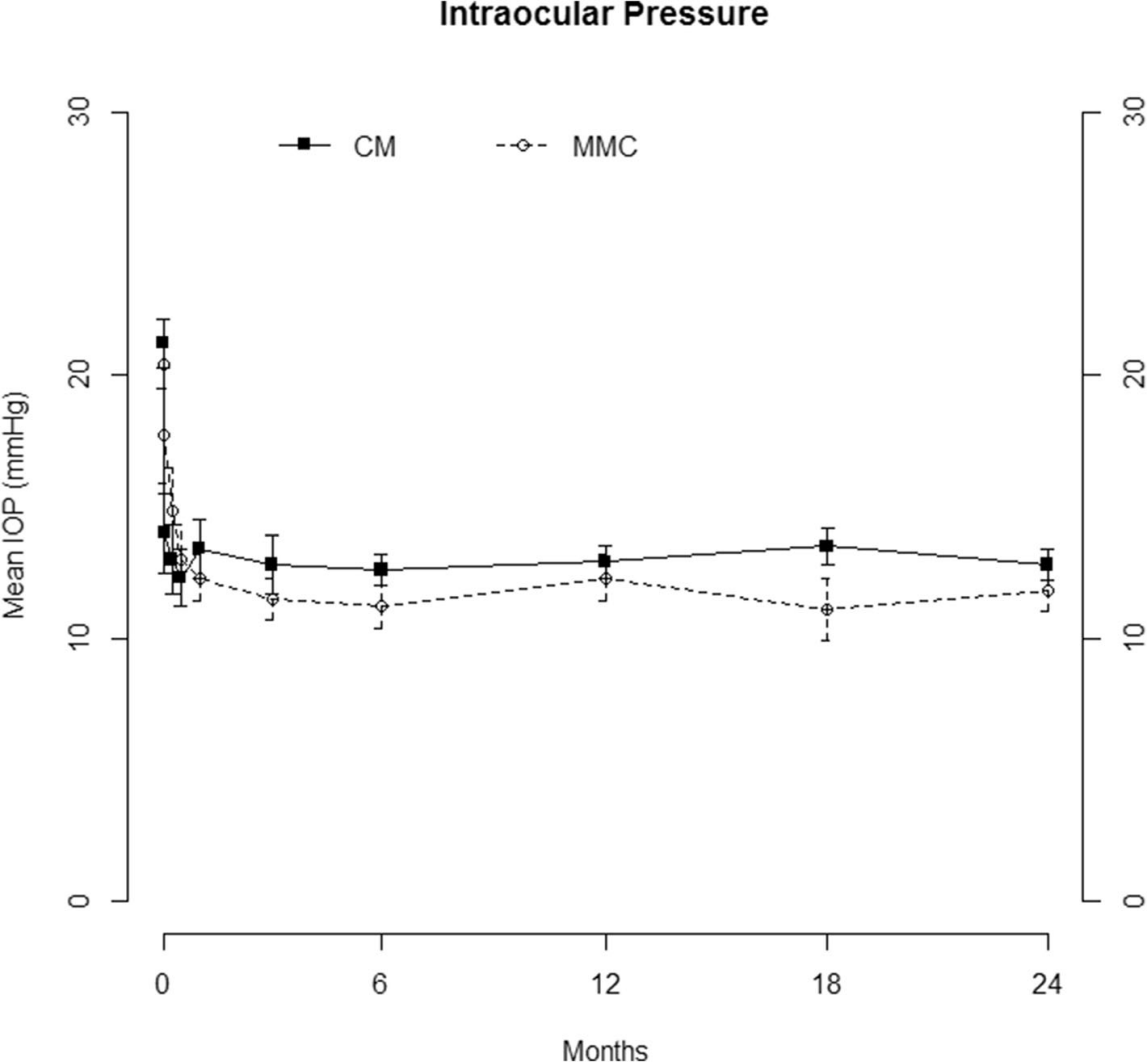
Mean intraocular pressure at each study visit in the collagen matrix (CM) and mitomycin-C (MMC) groups (*p* > 0.05 comparing CM with MMC at all the time points). The error bars represent standard deviations. Abbreviations: IOP = Intraocular Pressure; MMC = Mitomycin C; CM = Collagen Matrix

**Fig. 3:**
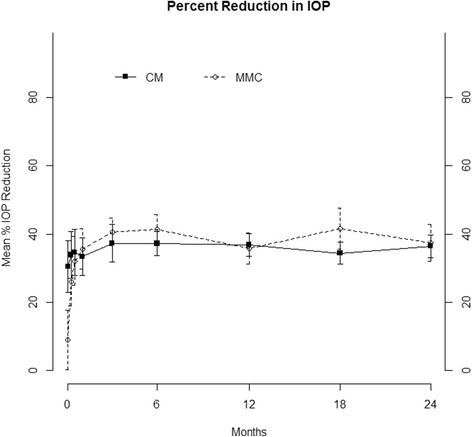
Mean percent reduction in intraocular pressure from the mean of the two baseline visits at each subsequent study visit in the collagen matrix (CM) and mitomycin-C (MMC) groups (*p* > 0.05 comparing CM with MMC at all the time points). The error bars represent standard deviations

**Fig. 4:**
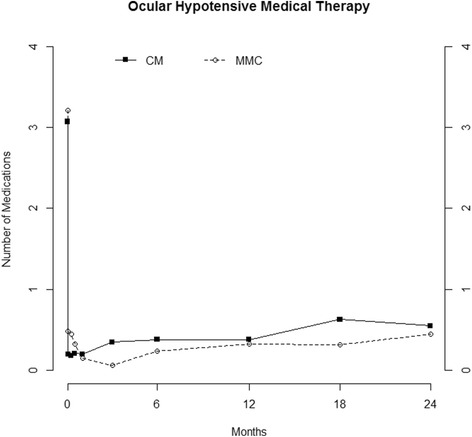
Mean number of ocular hypotensive medications at the baseline visit prior to surgery and each subsequent study visit in the collagen matrix (CM) and mitomycin-C (MMC) groups (*p* < 0.05 by Wilcoxon rank sum test at days 1 and 7).

IOP (Fig. 2, Additional file 1: Table S1) and percent reduction in IOP from baseline (Fig. 3, Additional file 2: Table S2) as well as mean number of ocular hypotensive medications (Fig. 4, Additional file 3: Table S3) were statistically similar for all time points. Visual acuity (Additional file 4: Table S4), visual field mean deviation, and rates of visual field change (Additional file 5: Table S5) were statistically similar between groups at all time points.

**Fig. 5:**
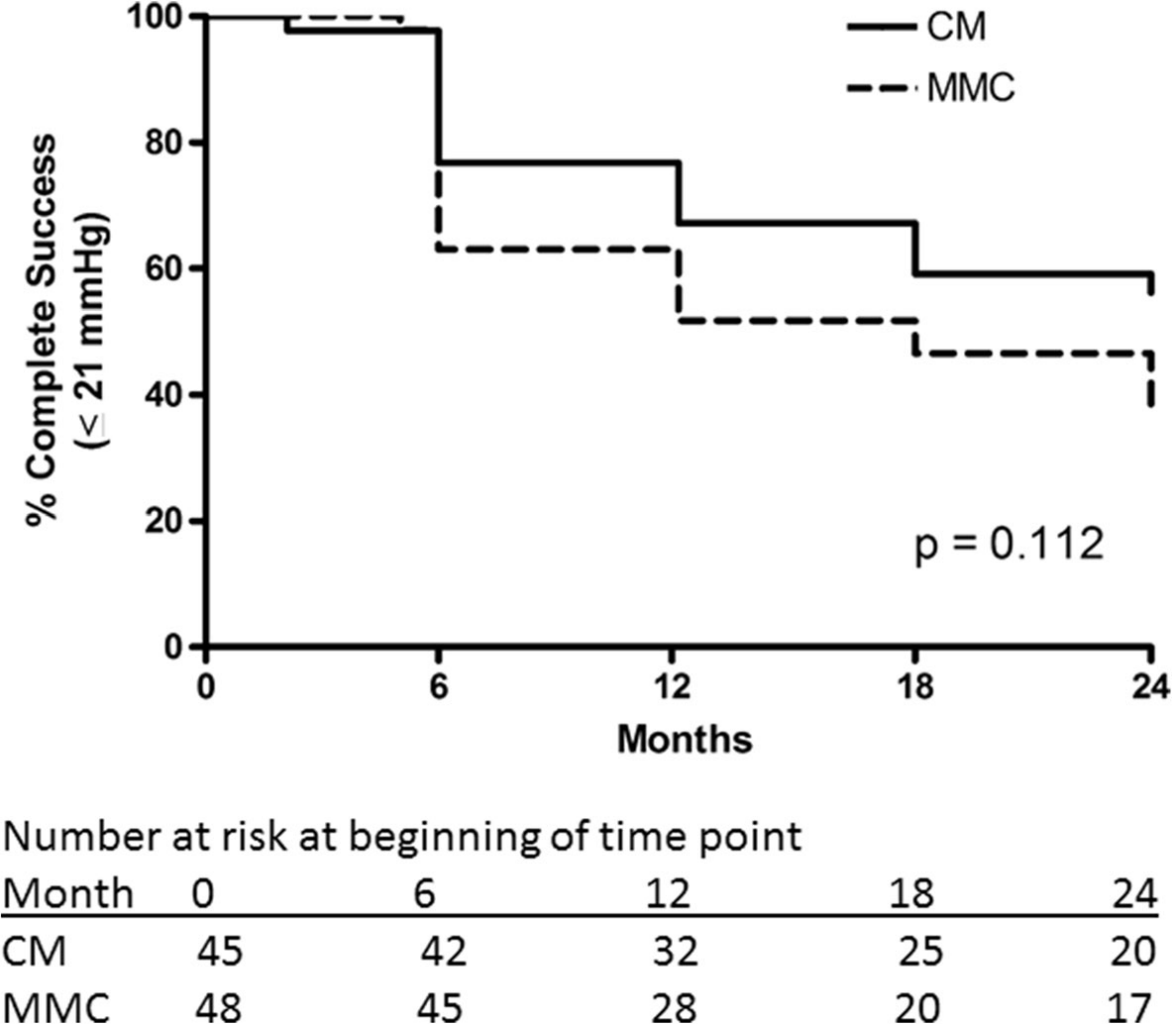
Kaplan-Meier curve demonstrating complete success rates in the collagen matrix (CM) and mitomycin-C (MMC) groups using the intraocular pressure criteria > 5 mmHg, ≤ 21 mmHg and 20% below the medicated baseline IOP

**Fig. 6:**
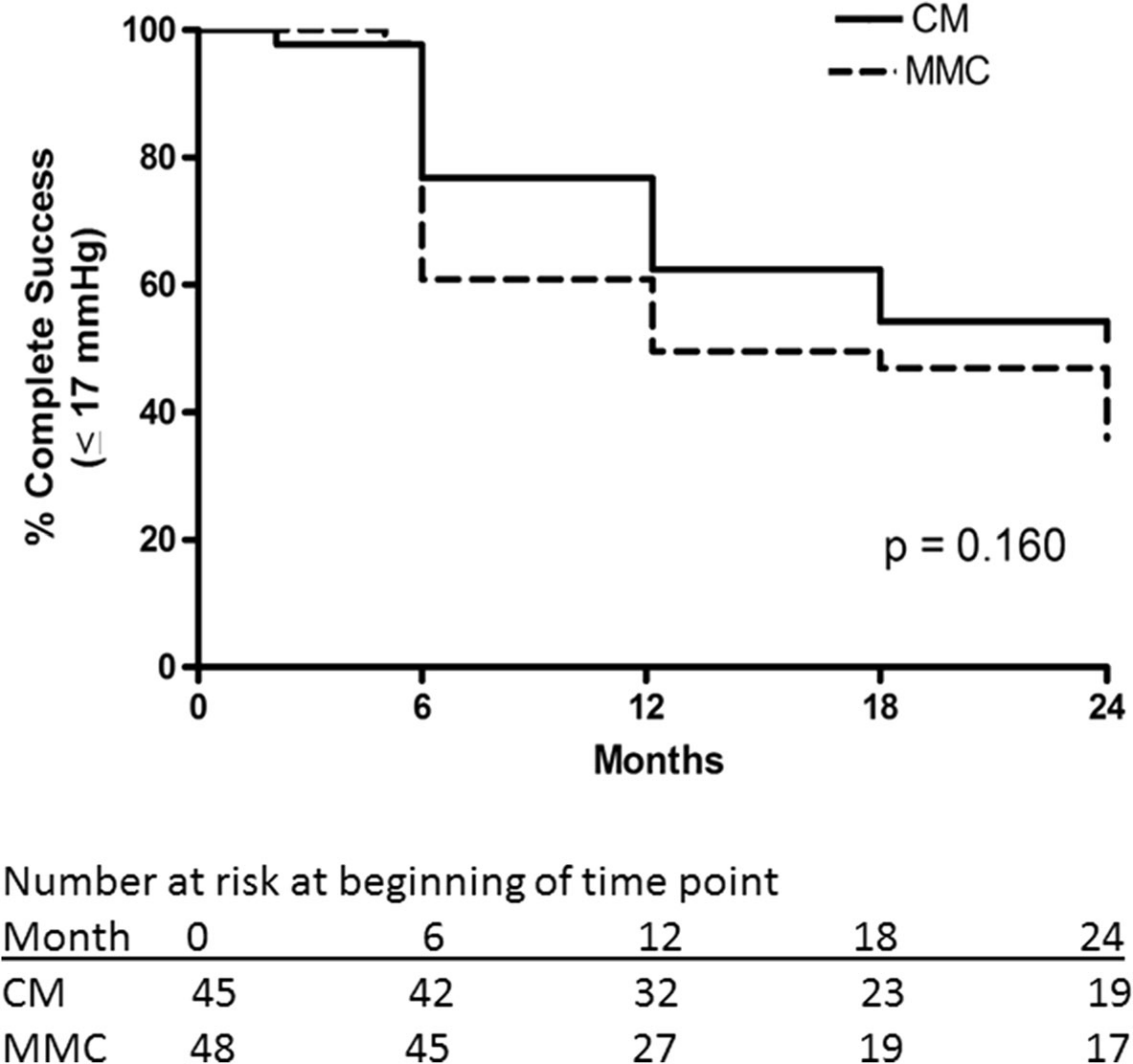
Kaplan-Meier curve demonstrating complete success rates in the collagen matrix (CM) and mitomycin-C (MMC) groups using the intraocular pressure criteria > 5 mmHg, ≤ 17 mmHg with a 20% below the medicated baseline IOP

Surgical success rates are summarized in Additional file 6: Table S6. Complete surgical success rates with cutoff criteria of IOP ≤21 mmHg (Fig. 5) or ≤17 mmHg (Fig. 6) along with ≥20% reduction below medicated baseline and IOP >5 mmHg were similar in both groups (*p* = 0.112 and 0.16, respectively). After exclusion of 10 subjects who underwent combined phacoemulsification and trabeculectomy, there remained no significant difference in success rates (*p* = 0.24 and 0.37 for IOP ≤21 and ≤17 mmHg, respectively). There were 3 subjects, all in the MMC group, in whom the numerical criterion for hypotony (IOP ≤5 mmHg on two consecutive study visits) was met, but in whom there were no structural or functional consequences of hypotony. After redefining these 3 subjects as surgical successes, there remained no significant difference between groups (*p* = 0.23 and 0.31 for IOP ≤21 and ≤17 mmHg, respectively).

**Fig. 7:**
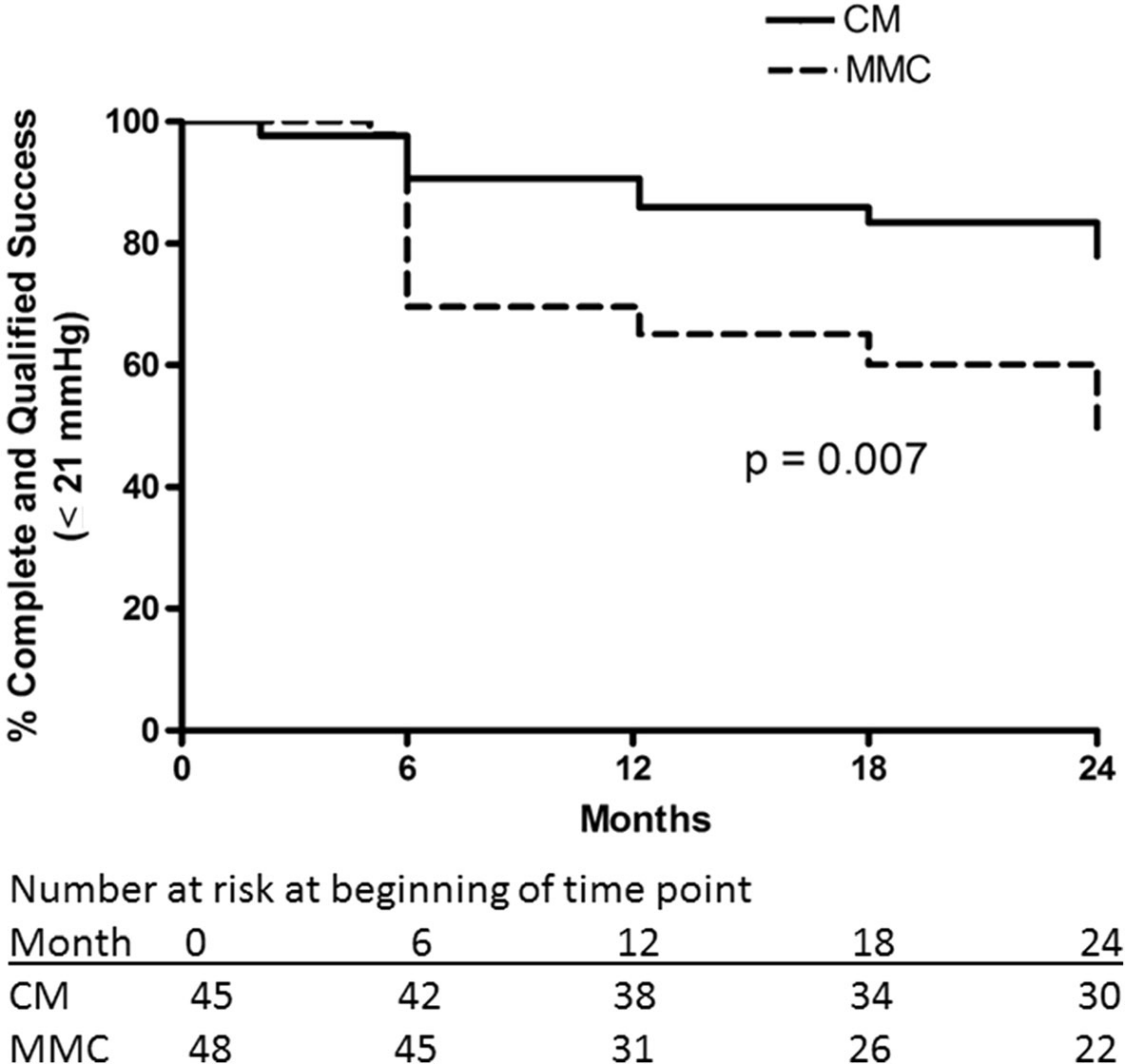
Kaplan-Meier curve demonstrating overall success rates in the collagen matrix (CM) and mitomycin-C (MMC) groups using the intraocular pressure criteria > 5 mmHg, ≤ 21 mmHg with a 20% below the medicated baseline IOP with or without the use of medication at the time of the study visit

**Fig. 8:**
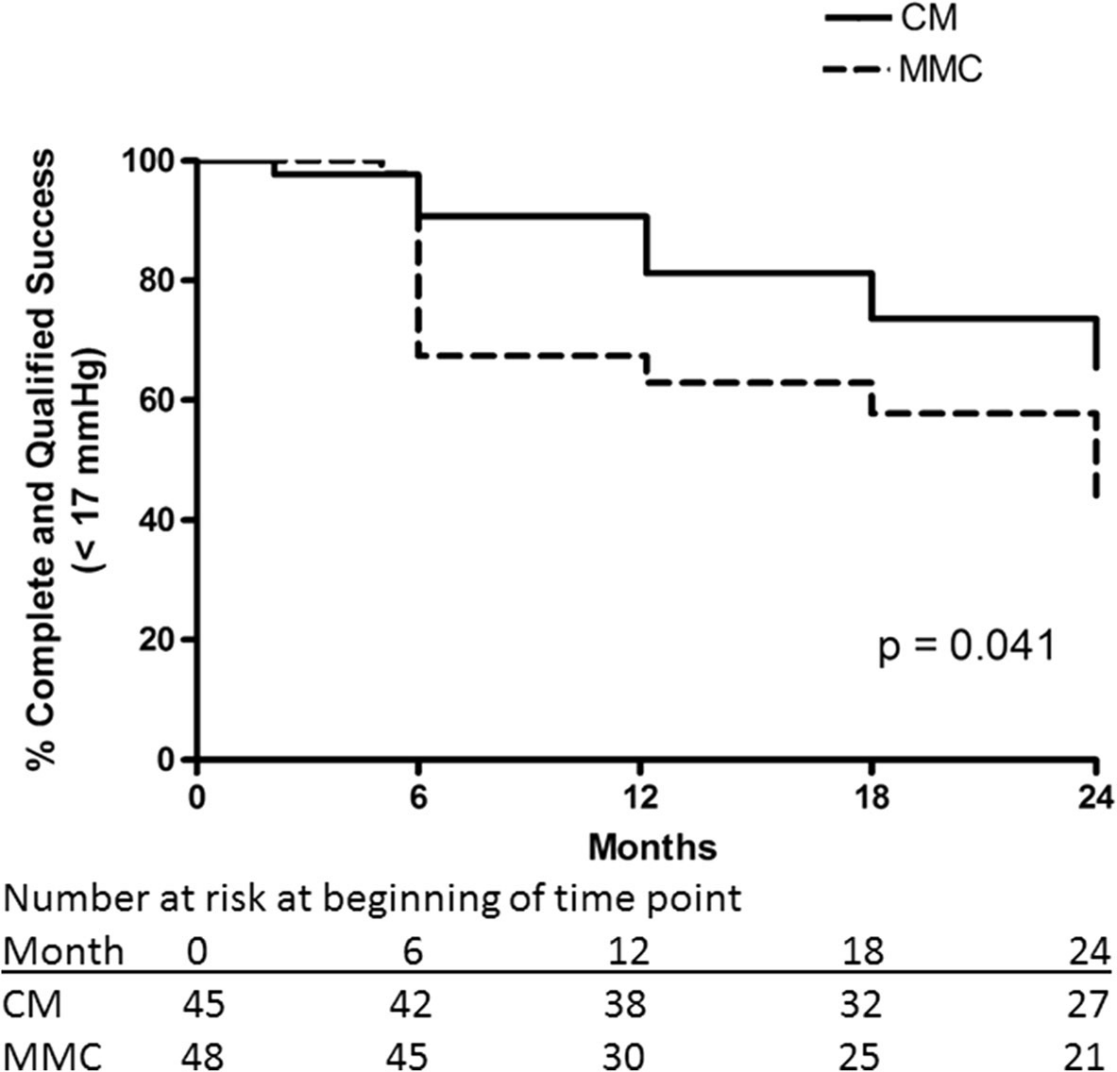
Kaplan-Meier curve demonstrating overall success rates in the collagen matrix (CM) and mitomycin-C (MMC) groups using the intraocular pressure criteria > 5 mmHg, ≤ 17 mmHg with a 20% below the medicated baseline IOP with or without the use of medication at the time of the study visit

The overall success rates (combined complete and qualified success, with medication) with the same criteria were significantly better in the CM group for IOP cutoff ≤21 mmHg (*p* = 0.007, Fig. 7) and IOP cutoff ≤17 mmHg (*p* = 0.041, Fig. 8). The difference remained statistically significant for IOP cutoff ≤ 21 mmHg after excluding the combined surgery eyes (*p* = 0.014) but not for IOP cutoff ≤ 17 (*p* = 0.087). After redefining the 3 subjects with hypotony based only on numerical criteria as successful, overall success rate remained significantly better in the CM group (*p* = 0.020 and 0.010, respectively, for IOP cutoff ≤ 21 and ≤ 17 mmHg). After adjusting for the occurrence of cataract surgery (prior to enrollment, combined with trabeculectomy or during the course of the follow-up period), overall success (cutoff ≤21 mmHg) remained significantly better in the CM group (*p* = 0.031), with other success outcomes being non-significant (complete success with cutoff ≤21 mmHg – *p* = 0.21, overall success with cutoff ≤17 mmHg – *p* = 0.11, complete success with cutoff ≤17 mmHg – *p* = 0.29). At each time point, numbers at risk in Figs. 5, 6, 7 and 8 may be less than the number of patients followed to that time point due to removal from the risk set for success in the Kaplan-Meier curves.

### Adverse events

Early, transient hypotony as well as associated complications such as hypotony maculopathy and serous choroidal detachment occurred more frequently in the CM group; however, the difference was not statistically significant (Table 3). Hypotony and its sequelae resolved in all of these subjects prior to the six-month study visit.

**Table 3:** Adverse events

Adverse event	Mitomycin-C (*n* = 48)	Collagen matrix (*n* = 45)	*p*-value (Fisher’s exact test)
Wound Leak (Within 30 Days of Surgery)	2	3	0.67
Bleb Leak (After 30 Days from Surgery)	1	0	0.99
Chalazion	1	0	0.99
Corneal Abrasion	0	1	0.48
Corneal Edema	2	1	0.99
Corneal Haze	0	1	0.48
Cystoid Macular Edema	1	0	0.99
Hyphema	1	0	0.99
Hypotony Maculopathy^a^	0	2	0.24
Iritis	0	1	0.48
Macular Scar	0	1	0.48
Choroidal Neovascularization	1	0	0.99
Serous Choroidal Detachment^a^	1	1	0.99
Photophobia	1	0	0.99
Ptosis	1	0	0.99
Uncontrolled IOP within 30 Days of	1	4	0.19

*IOP* intraocular pressure

^a^All cases of hypotony maculopathy and serous choroidal detachment occurred prior to the six-month visit

**Fig. 9:**
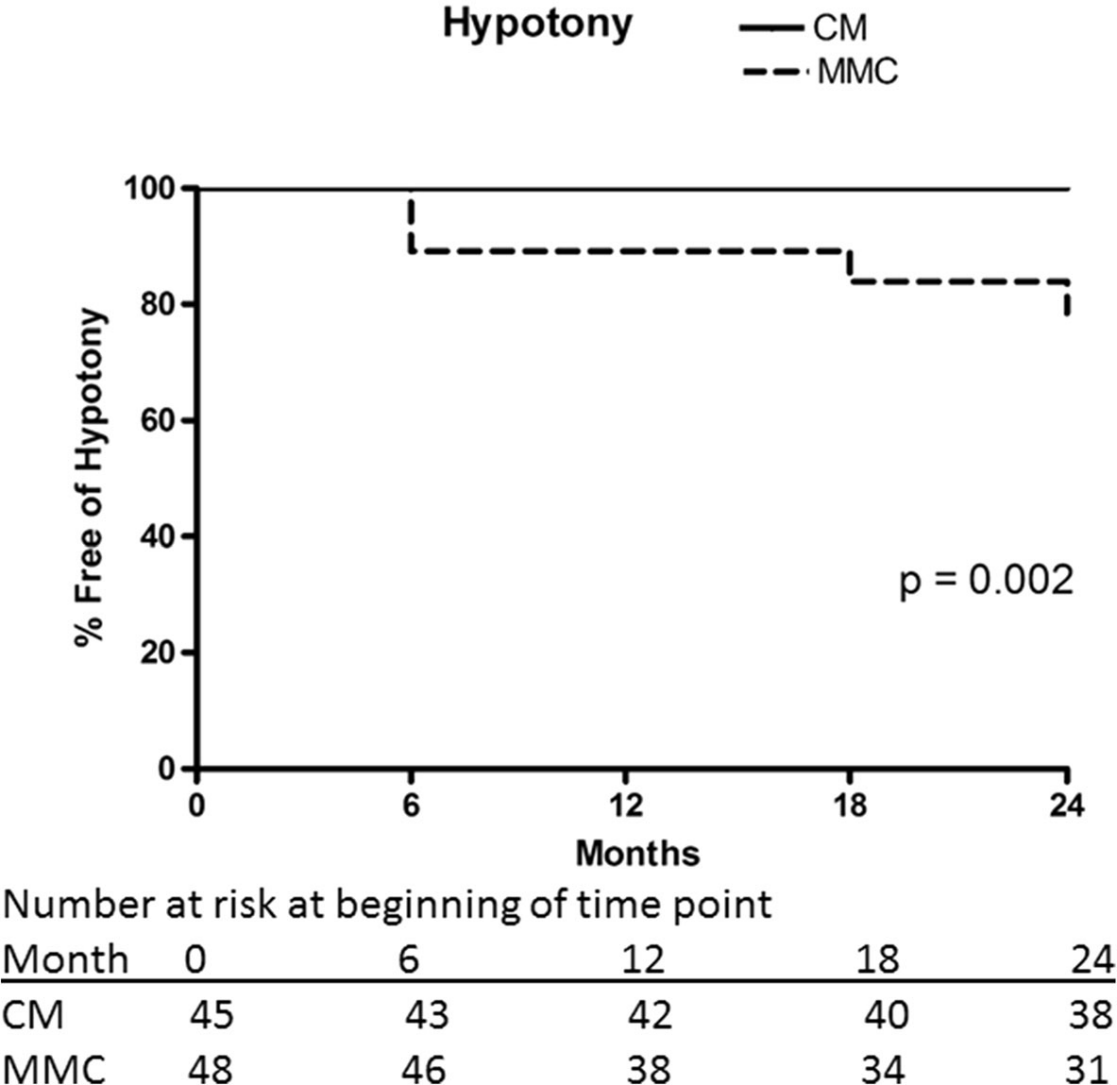
Kaplan-Meier curve demonstrating hypotony-free rates in the collagen matrix (CM) and mitomycin-C (MMC) groups using the intraocular pressure criterion ≤ 5 mmHg on two consecutive visits, starting at the 6-month visit. If IOP was ≤ 5 mmHg at the final study visit, the patient was classified as having met the definition of hypotony. The risk of hypotony was significantly higher in the MMC group (*p* = 0.002)

Nine subjects in the MMC group and none of the subjects in the CM group met the criteria for persistent hypotony as defined in the Adverse Events subsection in the Methods section above. The risk of persistent hypotony was significantly higher for the MMC group compared to the CM group (*p* = 0.002, Kaplan-Meier analysis, log rank test, Fig. 9). Six eyes with persistent hypotony had functional damage manifested as a reduction in visual acuity of at least 2 lines attributable to hypotony. Three eyes with IOP ≤5 mmHg on two consecutive visits, meeting the study definition for persistent hypotony had no structural or functional consequences attributable to hypotony. All 3 subjects were in the MMC group. When these subjects were re-classified as not meeting the criteria for persistent hypotony, the risk of persistent hypotony remained significantly higher in the MMC group (*p* = 0.022). Similarly, when the subjects who underwent phacoemulsification combined with trabeculectomy were excluded, the difference remained statistically significant (*p* = 0.002).

Other adverse events are presented in Table 3. There were no significant differences in the occurrence of any other adverse event. Transconjunctival needle revision (TCNR) was performed only one time in each of 4 subjects in the MMC group (with adjunctive 5-FU in 2 eyes and with MMC in 2 eyes) and 2 subjects in the CM group (twice in one subject – once without an antifibrotic agent and once with 5-FU) and once with MMC in one subject who underwent drainage device shunt surgery and failed on that basis prior to the one-year study visit). The need to undergo TCNR did not constitute an event that was defined as resulting in surgical failure. Additional glaucoma surgery (drainage device implantation) was performed in 3 subjects in the MMC group and 2 in the CM group.

## Discussion

Trabeculectomy remains the most commonly performed incisional glaucoma operation, particularly in eyes for which a low target IOP is desired. Refinements to the procedure aimed at improving the success rate and reducing the incidence of complications would be of value. This randomized clinical trial demonstrated two-year outcomes of trabeculectomy or combined phacoemulsification and trabeculectomy with adjunctive MMC are similar to those observed with adjunctive use of CM with respect to Kaplan-Meier complete success rates and mean IOP; however, there was a significantly lower incidence of sustained hypotony in the CM group with both simple numerical criteria or with more stringent criteria that require structural or functional consequences of the low IOP.

The complete and qualified success rates observed in the MMC group were similar to those reported in other randomized clinical trials. For example, the three-year complete success rate in the Tube Versus Trabeculectomy (TVT) study, defined as IOP ≤21 mmHg and 20% below medicated baseline and >5 mmHg without medications was 40% and an additional 26% were qualified successes [10]. With the same criteria for success in this study 38 and 56% of subjects achieved complete success and an additional 11 and 22% achieved qualified success in the MMC and CM groups, respectively. Patients in TVT, however, had undergone previous conjunctival and/or cataract surgery whereas only eyes without prior conjunctival surgery were eligible in the present study. Another difference between the two studies is the longer duration of follow-up in the TVT study; however, inspection of the Kaplan-Meier survival curves included in the 3-year TVT report suggests there was little difference in success rates between years two and three.

The Collaborative Initial Glaucoma Treatment Study (CIGTS) was a clinical trial in which eyes without previous surgery were randomized to initial treatment with medical therapy or trabeculectomy [11]. The mean baseline IOP was substantially higher in the surgery arm of CIGTS than in the present study, 27.4 ± 5.7 mmHg compared to 20.4 ± 6.0 mmHg in the MMC group and 21.9 ± 7.4 mmHg in the CM group. This is at least in part due to the fact none of the CIGTS patients were on medical therapy and all of the patients in the current study were on at least one medication at baseline. Although success rates based on IOP criteria were not reported for CIGTS, the mean IOP in the surgery arm was 14.4 ± 4.3 mmHg at 3 years compared to 11.8 ± 5.2 in the MMC group and 12.8 ± 3.7 in the CM group at 2 years [11]. Antifibrotic therapy was limited to the use of intraoperative and/or postoperative 5-fluorouracil, possibly accounting for the higher mean IOP at 3 years in CIGTS.

Other investigators have previously compared the results of trabeculectomy with MMC with trabeculectomy with the Ologen CM. In one small, randomized clinical trial with 15 subjects in each group and one-year follow-up data, the mean IOP was significantly higher and the success rate lower in the CM group versus MMC [12]. Those investigators, however, used a disc with a thickness of 2 mm and diameter of 6 mm compared to the 1 × 12 mm diameter disc used in this study. Late hypotony was observed in 7% of subjects in the CM group and 20% of subjects in the MMC group; however, no further details were reported. Histopathology of implants explanted from human eyes with failed trabeculectomies in that study disclosed the presence of fibroblasts, myofibroblasts, and fibronectin within the implant and enclosure of the implants by a collagenous pseudocapsule [12]. An earlier randomized trial of 10 subjects in each group conducted by the same group disclosed lower success rates in the CM group after one-year follow-up; however, they observed more avascular areas in the blebs in the MMC group [13]. It is possible the thicker, 2 mm CM disc results in excessive fibrosis over the scleral flap, resulting in lower success rates.

A retrospective study with 33 subjects in each group similarly had worse outcomes in the CM group [14]. In the same study, anterior segment OCT imaging disclosed no difference in bleb morphology between the two groups at 30 and 60 days; however, at 90 days, bleb height was lower with CM but there was no difference in bleb surface area. In a separate publication, the same investigators reported the Ologen CM implant was visible in ASOCT images in one third of eyes at 90 days [15]. Conversely, Marey, et al. [16] and Cillino, et al. [17] reported similar efficacy at 12 and 24 months of follow-up, respectively, in randomized clinical trials of MMC versus CM. The 2 mm thick CM disc was used in both studies. Senthil, et al. [18] also found similar outcomes at six months in a randomized clinical trial; however, there was a higher incidence of hyphema in the CM group that the authors attributed to the use of loose scleral flap sutures allowing entry of blood from the surgical incisions into the anterior chamber.

Two meta-analyses of randomized clinical trials comparing adjunctive use of MMC versus the Ologen CM for trabeculectomy have been published. One encompasses 224 participants in 6 clinical trials [19] and the other 227 eyes in 7 clinical trials [9]. Both disclosed no significant differences in outcomes; however, the authors of the latter study cautioned that the evidence is limited and stated additional randomized controlled trials are needed.

Studies in which MMC soaked Ologen CM implants were utilized at the time of trabeculectomy demonstrated no advantage to trabeculectomy with adjunctive MMC alone [20]. It is likely the utility of the CM device is dependent to some degree on fibroblast proliferation into the implant; therefore, the use of MMC in the sponge may obviate its benefits.

A small retrospective comparative case series of eyes that underwent trabeculectomy combined with the ExPress shunt showed that eyes in which the Ologen CM was used alone had worse IOP outcomes, a higher reoperation rate, and a higher rate of bleb leaks compared to eyes that underwent trabeculectomy with Ologen CM with adjunctive 5-FU or eyes that underwent trabeculectomy with MMC alone [21]. The investigators used the 6- × 2-mm CM implant in this study. A larger retrospective comparative case series of 49 eyes of 37 patients that underwent trabeculectomy with the ExPress shunt and Ologen CM had similar outcomes to those observed in 50 eyes of 48 patients which underwent trabeculectomy with the ExPress shunt with MMC [22].

Techniques with the use of CM during trabeculectomy have improved as surgeons have developed more experience with the device and disseminated knowledge regarding the optimization of its use. Specifically, due to the pressure of the CM disc on the scleral flap, scleral flap sutures must be adjusted to have low tension when the CM device is implanted. When the scleral flap sutures are tight, egress of aqueous along the edges of the scleral flap is insufficient, increasing the risk of failure. Laser suture lysis of scleral flap sutures is difficult with the CM material in place; therefore, releasable sutures may be preferable when using CM. The thickness of the CM disc may be important. We believe the thicker, 2-mm thick disc may result in the formation of excessive fibrotic tissue overlying the scleral flap and may result in the application of excessive pressure on the scleral flap resulting in insufficient egress of aqueous. Additionally, the conjunctival closure may be under more tension in some eyes when the thicker CM implant is utilized, possibly increasing the risk of the development of a wound leak.

We observed a significantly higher incidence of late hypotony in the MMC group. It is our impression the conjunctiva is thicker and less avascular in eyes that underwent trabeculectomy with CM, reducing the risk of late hypotony. Indeed, the use of Ologen CM implantation to treat post-trabeculectomy hypotony has been reported [23]. Paradoxically, frank hypotony maculopathy occurred as an adverse event in two cases in the CM group. Both of these were early and resolved by 14 and 90 days.

Limitations of this study include the fact the surgeons, of necessity, were not masked to treatment assignment. Subsequent decisions regarding reoperation for glaucoma and initiation of medical therapy could have been subject to bias. Very few subjects underwent reoperation for glaucoma, only 3 in the MMC group and 2 in the CM group. Although there was no consensus on this issue in the World Glaucoma Association Guidelines on Design and Reporting of Glaucoma Surgical Trials [24], needle revisions were not classified as additional glaucoma surgery in this study. Very few subjects underwent needle revision, and there was no significant difference in the utilization of this procedure between groups.

An additional limitation of this study relates to the fact subjects were permitted to undergo combined phacoemulsification and trabeculectomy. Although there was no significant difference in the number of subjects who underwent combined surgery, there were 4 such subjects in the MMC group compared to 6 in the CM group. Additionally, of the 3 patients with angle closure glaucoma in the MMC group, 2 underwent combined surgery and trabeculectomy. Of the 2 in CM, 1 underwent combined surgery and trabeculectomy. The impact of cataract surgery on IOP outcomes could have been larger in these patients. Due to the small number of subjects who underwent combined surgery, it is not possible to meaningfully analyze outcomes in that subgroup. Unlike the impact of combining phacoemulsification with minimally invasive glaucoma surgeries, IOP outcomes tend to be worse in eyes that undergo combined phacoemulsification and trabeculectomy versus trabeculectomy alone [25]. Since it is well established, however, that cataract surgery alone can result in IOP reduction, an ancillary analysis of success rates was performed after excluding all 10 subjects who underwent combined surgery as part of a *post-hoc* confirmatory analysis. This did not change any of the basic statistical conclusions with respect to the IOP-lowering efficacy of the two treatment assignments except for the overall success rate (success with or without medication) with an IOP cutoff of ≤ 17 mmHg. That definition of success was found to be significantly better in the CM group for the entire cohort; however, after exclusion of the combined phacoemulsification and trabeculectomy cases, the *p*-value for the difference between groups was no longer statistically significant at *p* = 0.087. A proportional hazards model with age, sex, and combined cataract surgery as covariates showed no statistically significant influence on the initial results regarding overall success rates for an IOP cutoff of 21 mmHg within the limit of our sample size except the difference in overall success rates for an IOP cutoff of 17 mmHg was no longer significantly different. Additionally, our sample size calculation was based on failure rates due to hypotony. We chose this outcome because this was a non-inferiority trial with respect to IOP reduction, as such, we did not anticipate significant differences in IOP between the two groups. Given the surgical technique is very similar between the two groups, aside from modulation of scarring, non-inferiority trials are often conducted to test whether one treatment arm offers a better safety profile [26]. Therefore, our results should not be used to drive conclusions regarding differences in IOP-lowering efficacy between the two groups. Future studies with longer follow-up and larger sample sizes are warranted for that purpose.

Significantly more subjects in the MMC group had had prior cataract surgery. Although these were performed via clear corneal incisions, using a proportional hazards model with age, sex, and prior cataract surgery as covariates we were not able to demonstrate a statistically significant impact of previous cataract surgery on the initial results regarding complete and overall success rates within the limit of our sample size except for overall success with an IOP cutoff of 17 mmHg, which became non-significantly different.

We followed the methodology of the Tube versus Trabeculectomy Trial with respect to using a numerical cutoff for hypotony as part of the definition of success [10]. This is also consistent with the Guidelines on Design and Reporting of Glaucoma Surgical Trials consensus document prepared by the World Glaucoma Association [24]. Three subjects with hypotony as defined by an IOP ≤5 mmHg did not have adverse structural or functional outcomes as a result. Sustained hypotony, defined as an IOP ≤5 mmHg on two consecutive visits beginning with the six-month visit, was a criterion for failure, and the incidence of hypotony was significantly higher in the MMC group with either the simple numerical criterion or a more conservative definition of hypotony that necessitated the presence of hypotony maculopathy, optic disc edema, choroidal detachment or a reduction in visual acuity along with an IOP ≤5 mmHg.

## Conclusions

In this two-year randomized clinical trial, no measured difference in complete success rates were observed between eyes that underwent trabeculectomy with adjunctive MMC versus implantation of the Ologen CM. The risk of sustained hypotony was significantly lower in the CM group. Additional clinical trials with longer follow up are needed.

## Additional files

Additional file 1: Table S1. Intraocular Pressure at Baseline and at Each Study Visit. (DOCX 20 kb)
Additional file 2: Table S2. Percent Reduction in Intraocular Pressure from Baseline and at Each Study Visit. (DOCX 24 kb)
Additional file 3: Table S3. Number of Glaucoma Medications in Use at Baseline. (DOCX 24 kb)
Additional file 4: Table S4. Visual Acuity. (DOCX 23 kb)
Additional file 5: Table S5. Visual Field Mean Deviation. (DOCX 21 kb)
Additional file 6: Table S6. Kaplan-Meier Complete and Overall Success Rates for IOP ≤ 21 and ≤ 17 mmHg. (DOCX 22 kb)

## Abbreviations

5-FU: 5-fluorouracil
CIGTS: Collaborative initial glaucoma treatment study
CM: Collagen matrix
IOP: Intraocular pressure
IRB: Institutional Review Board
MMC: Mitomycin C
TCNR: Trans-conjunctival needle revision
TVT: Tube versus trabeculectomy study
