# Supplementary material for: Collagen matrix vs mitomycin-C in trabeculectomy and combined phacoemulsification and trabeculectomy: a randomized controlled trial
Source: BMC Ophthalmol. 2016 Dec 29;16:217. doi: 10.1186/s12886-016-0393-z (PMC5200961; doi:10.1186/s12886-016-0393-z)
Supplement: Additional file 1: Table S1. — Intraocular Pressure at Baseline and at Each Study Visit. (DOCX 20 kb) [file 12886_2016_393_MOESM1_ESM.docx]

**Additional file 1: Table S1. Intraocular Pressure at Baseline and at Each Study Visit**

|  |  |  |  |  |
| --- | --- | --- | --- | --- |
|  | IOP |  |  |  |
|  |  |  |  |  |
|  |  | MMC | CM | p-value, t-test, not adjusted for multiple testing |
| Baseline | N | 48 | 45 |  |
|  | MEAN (SD) | 20.4 (6.0) | 21.2 (6.1) | 0.49 |
|  | median (IQR) | 19.8 (16.0 - 23.6) | 19.0 (17.0 - 26.0) |  |
|  | SEM | 0.9 | 0.9 |  |
| Note: p-values take into account any difference in standard deviation in the two groups. |  |  |  | p-value, analysis of covariance adjusting for baseline IOP, not adjusted for multiple testing |
| 1 day | n | 48 | 45 |  |
|  | mean (sd) | 17.7 (12.8) | 14.0 (10.2) | 0.09 |
|  | median (IQR) | 16.0 (8.0 - 24.3) | 12.5 (6.5 – 17.5) |  |
|  | SEM | 1.8 | 1.5 |  |
|  |  |  |  |  |
| 7 day | n | 47 | 44 |  |
|  | mean (sd) | 14.8 (11.8) | 13.0 (8.4) | 0.32 |
|  | median (IQR) | 12.5 (7.5 – 20.0) | 12.0 (6.0 - 19.3) |  |
|  | SEM | 1.7 | 1.3 |  |
|  |  |  |  |  |
| 14 day | n | 48 | 42 |  |
|  | mean (sd) | 13.0 (8.9) | 12.3 (6.9) | 0.70 |
|  | median (IQR) | 10.5 (6.8 - 17.5) | 11.8 (7.0 – 16.0) |  |
|  | SEM | 1.3 | 1.1 |  |
|  |  |  |  |  |
| 30 day (1 month) | n | 48 | 45 |  |
|  | mean (sd) | 12.3 (6.4) | 13.4 (7.1) | 0.47 |
|  | median (IQR) | 12.0 (7.8 - 16.0) | 12.0 (9.0 - 16.0) |  |
|  | SEM | 0.9 | 1.1 |  |
|  |  |  |  |  |
| 90 day (3 months) | n | 48 | 44 |  |
|  | mean (sd) | 11.5 (5.2) | 12.8 (7.4) | 0.43 |
|  | median (IQR) | 12.0 (7.0 - 15.0) | 11.0 (8.5 – 14.8) |  |
|  | SEM | 0.8 | 1.1 |  |
|  |  |  |  |  |
| 180 day (6 months) | n | 45 | 44 |  |
|  | mean (sd) | 11.2 (5.5) | 12.6 (3.8) | 0.23 |
|  | median (IQR) | 11.0 (7.5 - 14.0) | 11.5 (10.0 - 15.0) |  |
|  | SEM | 0.8 | 0.6 |  |
|  |  |  |  |  |
| 365 (1 year) | n | 41 | 42 |  |
|  | mean (sd) | 12.3 (5.6) | 12.9 (3.9) | 0.76 |
|  | median (IQR) | 12.5 (8.0 - 16.0) | 13.0 (10.0 - 14.0) |  |
|  | SEM | 0.9 | 0.6 |  |
|  |  |  |  |  |
| 548 (18 month) | n | 39 | 39 |  |
|  | mean (sd) | 11.1 (7.5) | 13.5 (4.1) | 0.14 |
|  | median (IQR) | 10.0 (7.0 – 15.5) | 13.0 (10.0 - 16.5) |  |
|  | SEM | 1.2 | 0.7 |  |
|  |  |  |  |  |
| 730 (2 year) | n | 37 | 38 |  |
|  | mean (sd) | 11.8 (5.2) | 12.8 (3.7) | 0.36 |
|  | median (IQR) | 13.0 (9.0 - 16.5) | 12.5 (10.0 - 16.0) |  |
|  | SEM | 0.8 | 0.6 |  |
|  |  |  |  |  |

**Abbreviations:**

**IOP=Intraocular Pressure; SD=Standard Deviation; IQR=Interquartile Range; SEM=Standard Error of the Mean**
