# Supplementary material for: Collagen matrix vs mitomycin-C in trabeculectomy and combined phacoemulsification and trabeculectomy: a randomized controlled trial
Source: BMC Ophthalmol. 2016 Dec 29;16:217. doi: 10.1186/s12886-016-0393-z (PMC5200961; doi:10.1186/s12886-016-0393-z)
Supplement: Additional file 2: Table S2. — Percent Reduction in Intraocular Pressure from Baseline and at Each Study Visit. (DOCX 24 kb) [file 12886_2016_393_MOESM2_ESM.docx]

**Additional file 2: Table S2. Percent Reduction in Intraocular Pressure from Baseline and at Each Study Visit**

|  |  |  |  |  |
| --- | --- | --- | --- | --- |
|  | IOP Percent reduction from baseline |  |  |  |
| Note: p-values take into account any difference in standard deviation in the two groups. |  | MMC | CM | p-value, analysis of covariance adjusting for baseline IOP, not adjusted for multiple testing |
| 1 day | n | 48 | 45 |  |
|  | mean (sd) | 9.0 (60.5) | 30.4 (51.2) | 0.09 |
|  | median (IQR) | 10.2 ( - 26.6 - 53.88) | 42.1 (1.8 - 64.4) |  |
|  | sem | 8.7 | 7.6 |  |
|  |  |  |  |  |
| 7 day | N | 47 | 44 |  |
|  | mean (sd) | 26.1 (48.5) | 33.9 (45.8) | 0.53 |
|  | median (IQR) | 27.7 ( - 16.1 - 67.3) | 45.9 ( - 17.0 - 71.5) |  |
|  | sem | 7.1 | 6.9 |  |
|  |  |  |  |  |
| 14 day | N | 48 | 42 |  |
|  | mean (sd) | 32.1 (49.0) | 34.6 (44.1) | 0.91 |
|  | median (IQR) | 41.8 ( 19.8 – 64.7) | 47.4 (12.5 – 64.9) |  |
|  | sem | 7.1 | 6.8 |  |
|  |  |  |  |  |
| 30 day (1 month) | N | 48 | 45 |  |
|  | mean (sd) | 35.6 (40.6) | 33.4 (39.3) | 0.60 |
|  | median (IQR) | 37.3 (22.6 - 66.7) | 40.8 (15.7 – 58.9) |  |
|  | sem | 5.9 | 5.5 |  |
|  |  |  |  |  |
| 90 day (3 months) | N | 48 | 44 |  |
|  | mean (sd) | 40.6 (28.4) | 37.3 (36.6) | 0.48 |
|  | median (IQR) | 47.0 (15.7 – 62.1) | 44.3 (22.4 - 60.4) |  |
|  | sem | 4.1 | 5.5 |  |
|  |  |  |  |  |
| 180 day (6 months) | N | 45 | 43 |  |
|  | mean (sd) | 41.4 (29.2) | 37.2 (23.7) | 0.23 |
|  | median (IQR) | 44.3 (26.9 - 64.3) | 42.9 (30.0 - 55.3) |  |
|  | sem | 4.3 | 3.6 |  |
|  |  |  |  |  |

| 365 (1 year) | n | 41 | 42 |  |
| --- | --- | --- | --- | --- |
|  | mean (sd) | 35.8 (29.5) | 36.8 (21.3) | 0.88 |
|  | median (IQR) | 32.8 (16.7 - 57.3) | 42.1 (26.9 - 51.1) |  |
|  | sem | 4.6 | 3.3 |  |
|  |  |  |  |  |
| 548 (18 month) | N | 39 | 39 |  |
|  | mean (sd) | 41.5 (37.6) | 34.4 (19.7) | 0.16 |
|  | median (IQR) | 45.9 (24.4 - 60.0) | 35.3 (19.1 – 48.2) |  |
|  | sem | 6.0 | 3.2 |  |
|  |  |  |  |  |
| 730 (2 year) | N | 37 | 38 |  |
|  | mean (sd) | 37.4 (33.0) | 36.4 (20.4) | 0.66 |
|  | median (IQR) | 38.1 (15.6 - 53.5) | 37.3 (29.7 – 47.9) |  |
|  | sem | 5.4 | 3.3 |  |

**Abbreviations:**

**IOP=Intraocular Pressure; SD=Standard Deviation; IQR=Interquartile Range; SEM=Standard Error of the Mean**
