# Supplementary material for: Collagen matrix vs mitomycin-C in trabeculectomy and combined phacoemulsification and trabeculectomy: a randomized controlled trial
Source: BMC Ophthalmol. 2016 Dec 29;16:217. doi: 10.1186/s12886-016-0393-z (PMC5200961; doi:10.1186/s12886-016-0393-z)
Supplement: Additional file 3: Table S3. — Number of Glaucoma Medications in Use at Baseline. (DOCX 24 kb) [file 12886_2016_393_MOESM3_ESM.docx]

**Additional file 3: Table S3. Number of Glaucoma Medications in Use at Baseline and Each Study Visit**

|  |  |  |  |  |
| --- | --- | --- | --- | --- |
|  | NUMBER OF GLAUCOMA MEDICATIONS |  |  |  |
|  |  |  |  |  |
|  |  | MMC | CM | p-value, t-test for means, Mann-Whitney U test for medians, not adjusted for multiple testing |
| Baseline | n | 48 | 44 |  |
|  | mean (sd) | 3.21 (1.13) | 3.07 (1.00) | 0.53 |
|  | median (IQR) | 3 (2-4) | 3 (2.5-3.5) | 0.48 |
|  | Range | 1-5 | 1-5 |  |
|  | SEM | .16 | .15 |  |
|  |  |  |  | p-value |
| 1 day | N | 48 | 44 |  |
|  | mean (sd) | .48 (1.05) | .20 (0.85) | 0.17 |
|  | median (IQR) | 0 (0-0) | 0 (0-0) | 0.040 |
|  | Range | 0-4 | 0-4 |  |
|  | SEM | .15 | .13 |  |
|  |  |  |  |  |
| 7 day | N | 47 | 44 |  |
|  | mean (sd) | .45 (0.93) | .18 (0.76) | 0.14 |
|  | median (IQR) | 0 (0-0) | 0 (0-0) | 0.036 |
|  | Range | 0-4 | 0-4 |  |
|  | SEM | .14 | .11 |  |
|  |  |  |  |  |
| 14 day | N | 48 | 43 |  |
|  | mean (sd) | .33 (.88) | .21 (.77) | 0.48 |
|  | median (IQR) | 0 (0-0) | 0 (0-0) | 0.44 |
|  | Range | 0-4 | 0-4 |  |
|  | SEM | .13 | .12 |  |
|  |  |  |  |  |
| 30 day (1 month) | N | 48 | 44 |  |
|  | mean (sd) | .15 (.50) | .20 (.76) | 0.67 |
|  | median (IQR) | 0 (0-0) | 0 (0-0) | 0.89 |
|  | Range | 0-2 | 0-4 |  |
|  | SEM | .07 | .12 |  |
|  |  |  |  |  |
| 90 day (3 months) | N | 47 | 43 |  |
|  | mean (sd) | .06 (.32) | .35 (.90) | 0.054 |
|  | median (IQR) | 0 (0-0) | 0 (0-0) | 0.054 |
|  | Range | 0-2 | 0-4 |  |
|  | SEM | .05 | .14 |  |
|  |  |  |  |  |
| 180 day (6 months) | N | 45 | 42 |  |
|  | mean (sd) | .24 (.74) | .38 (1.01) | 0.47 |
|  | median (IQR) | 0 (0-0) | 0 (0-0) | 0.47 |
|  | Range | 0-3 | 0-4 |  |
|  | SEM | .11 | .16 |  |
|  |  |  |  |  |
| 365 (1 year) | N | 43 | 42 |  |
|  | mean (sd) | .33 (.78) | .38 (.99) | 0.77 |
|  | median (IQR) | 0 (0-0) | 0 (0-0) | 0.89 |
|  | Range | 0-3 | 0-4 |  |
|  | SEM | .12 | .15 |  |
|  |  |  |  |  |
| 548 (18 months) | N | 41 | 38 |  |
|  | mean (sd) | .32 (.69) | .63 (1.15) | 0.15 |
|  | median (IQR) | 0 (0-0) | 0 (0-1) | 0.27 |
|  | Range | 0-3 | 0-4 |  |
|  | SEM | .11 | .19 |  |
|  |  |  |  |  |
| 730 (2 years) | N | 40 | 38 |  |
|  | mean (sd) | .45 (.81) | .55 (1.03) | 0.63 |
|  | median (IQR) | 0 (0-0) | 0 (0-1) | 0.83 |
|  | range | 0-3 | 0-4 |  |
|  | SEM | .13 | .17 |  |

**Abbreviations: SD=Standard Deviation; IQR=Interquartile Range; SEM=Standard Error of the Mean**
