# Supplementary material for: Collagen matrix vs mitomycin-C in trabeculectomy and combined phacoemulsification and trabeculectomy: a randomized controlled trial
Source: BMC Ophthalmol. 2016 Dec 29;16:217. doi: 10.1186/s12886-016-0393-z (PMC5200961; doi:10.1186/s12886-016-0393-z)
Supplement: Additional file 4: Table S4. — Visual Acuity. (DOCX 23 kb) [file 12886_2016_393_MOESM4_ESM.docx]

**Additional file 4: Table S4. Visual Acuity**

|  |  |  |  |  |
| --- | --- | --- | --- | --- |
|  | VA (LOGMAR) |  |  |  |
|  |  |  |  |  |
| Note: p-values take into account any difference in standard deviation in the two groups. |  | MMC | CM | p-value, t-test, not adjusted for multiple testing |
| Baseline | n | 45 | 42 |  |
|  | mean (sd) | .18 (.26) | .17 (.23) | .81 |
|  | median (IQR) | .10 (0 – .21) | .10 (0 – .30) |  |
|  | sem | 0.04 | .04 |  |
|  |  |  |  | p-value, analysis of covariance adjusting for baseline VA, not adjusted for multiple testing |
| 1 day | N | 45 | 43 |  |
|  | mean (sd) | .41 (.33) | .38 (.29) | 0.94 |
|  | median (IQR) | .40 (.18 - .54) | .30 (.18 - .54) |  |
|  | sem | .05 | .04 |  |
|  |  |  |  |  |
| 7 day | N | 42 | 41 |  |
|  | mean (sd) | .29 (.30) | .28 (.24) | 0.66 |
|  | median (IQR) | .18 (.10 - .40) | .18 (.10 - .40) |  |
|  | sem | .05 | .04 |  |
|  |  |  |  |  |
| 14 day | N | 43 | 38 |  |
|  | mean (sd) | .31 (.34) | .23 (.19) | 0.16 |
|  | median (IQR) | .18 (.10 - .40) | .18 (.10 - .30) |  |
|  | sem | .05 | .03 |  |
|  |  |  |  |  |
| 30 day (1 month) | N | 46 | 42 |  |
|  | mean (sd) | .32 (.32) | .23 (.21) | 0.10 |
|  | median (IQR) | .18 (.10 - .48) | .18 (.10 - .30) |  |
|  | sem | .05 | .03 |  |
|  |  |  |  |  |

| 90 day (3 months) | N | 40 | 41 |  |
| --- | --- | --- | --- | --- |
|  | mean (sd) | .22 (.31) | .18 (.22) | 0.25 |
|  | median (IQR) | .10 (0 - .30) | .10 (0 - .30) |  |
|  | sem | .05 | .03 |  |
|  |  |  |  |  |
| 180 day (6 months) | N | 35 | 37 |  |
|  | mean (sd) | .16 (.19) | .17 (.23) | 0.51 |
|  | median (IQR) | .10 (0 - .30) | .10 (0 - .18) |  |
|  | sem | .03 | .04 |  |
|  |  |  |  |  |
| 365 (1 year) | N | 27 | 30 |  |
|  | mean (sd) | .19 (.29) | .16 (22) | 0.22 |
|  | median (IQR) | .10 (0 - .30) | .10 (0 - .18) |  |
|  | sem | .06 | .04 |  |
|  |  |  |  |  |
| 548 (18 months) | N | 35 | 38 |  |
|  | mean (sd) | .20 (.25) | .20 (.26) | 0.76 |
|  | median (IQR) | .10 (.10 - .30) | .14 (0 - .30) |  |
|  | sem | .04 | .04 |  |
|  |  |  |  |  |
| 730 (2 years) | N | 33 | 36 |  |
|  | mean (sd) | .16 (.18) | .19 (.25) | 0.87 |
|  | median (IQR) | .10 (0 - .30) | .10 (0 - .30) |  |
|  | Sem | .03 | .04 |  |
|  |  |  |  |  |

**Abbreviations:**

**Log MAR=Logarithm of the Minimum Angle of Resolution; SD=Standard Deviation; IQR=Interquartile Range; SEM=Standard Error of the Mean**
