# Supplementary material for: Collagen matrix vs mitomycin-C in trabeculectomy and combined phacoemulsification and trabeculectomy: a randomized controlled trial
Source: BMC Ophthalmol. 2016 Dec 29;16:217. doi: 10.1186/s12886-016-0393-z (PMC5200961; doi:10.1186/s12886-016-0393-z)
Supplement: Additional file 5: Table S5. — Visual Field Mean Deviation. (DOCX 21 kb) [file 12886_2016_393_MOESM5_ESM.docx]

**Additional file 5: Table S5. Visual Field Mean Deviation**

|  |  |  |  |  |
| --- | --- | --- | --- | --- |
|  | VF-MEAN DEVIATION |  |  |  |
|  |  |  |  |  |
|  |  | MMC | CM | p-value, t-test, not adjusted for multiple testing |
| Baseline | n | 45 | 45 |  |
|  | mean (sem) | -14.1 (10.7) | -12.9 (8.6) | 0.55 |
|  | median (IQR) | -14.4 (-23.3 to -6.4) | -14.1 (-19.9 to -5.8) |  |
|  | sem | 1.6 | 1.3 |  |
|  |  |  |  |  |
|  |  |  |  | p-value, analysis of covariance adjusting for baseline VF, not adjusted for multiple testing |
| 180 day (6 months) | n | 38 | 41 |  |
|  | mean (sem) | -14.6 (8.3) | -11.4 (9.0) | 0.32 |
|  | median (IQR) | -14.4 (-21.9 to -8.4) | -10.3 (-17.7 to -3.7) |  |
|  | sem | 1.3 | 1.4 |  |
|  |  |  |  |  |
| 365 (1 year) | n | 39 | 41 |  |
|  | mean (sem) | -15.8 (9.3) | -13.2 (9.3) | 0.31 |
|  | median (IQR) | -15.5 (-24.3 to -9.5) | -13.5 (-20.0 to -4.7) |  |
|  | sem | 1.5 | 1.5 |  |
|  |  |  |  |  |
| 548 (18 months) | n | 36 | 35 |  |
|  | mean (sem) | -16.0 (8.5) | -12.0 (9.3) | 0.47 |
|  | median (IQR) | -15.2 (-22.2 to -10.0) | -10.1 (-17.7 to -4.0) |  |
|  | sem | 1.4 | 1.6 |  |
|  |  |  |  |  |
| 730 (2 years) | n | 33 | 32 |  |
|  | mean (sem) | -19.2 (16.6) | -14.1 (8.9) | 0.31 |
|  | median (IQR) | -18.9 (-24.3 to -10.5) | -14.4 (-20.0 to -6.4) |  |
|  | sem | 2.9 | 1.6 |  |
|  |  |  |  |  |
| Slope (se) from random effects model* |  | -2.15 (1.08) | -0.72 (1.05) | 0.34 |
| Slope (se) from random effects model** |  | -1.81 (0.65) | -0.42 (0.65) | 0.13 |

* Slope is population averaged change per year from a mixed effects model with person as the random effect, group and time and group by time interaction as fixed effects. Model adjusts for baseline value, but does not include baseline in calculation of slopes. P-value from interaction test.

**Slope is population averaged change per year from a mixed effects model with person as the random effect, group and time and group by time interaction as fixed effects. Model adjusts for baseline value, but includes baseline in calculation of slopes. P-value from interaction test.

**Abbreviations:**

**SD=Standard Deviation; IQR=Interquartile Range; SEM=Standard Error of the Mean**
