# Supplementary material for: Collagen matrix vs mitomycin-C in trabeculectomy and combined phacoemulsification and trabeculectomy: a randomized controlled trial
Source: BMC Ophthalmol. 2016 Dec 29;16:217. doi: 10.1186/s12886-016-0393-z (PMC5200961; doi:10.1186/s12886-016-0393-z)
Supplement: Additional file 6: Table S6. — Kaplan-Meier Complete and Overall Success Rates for IOP ≤ 21 and ≤ 17 mmHg. (DOCX 22 kb) [file 12886_2016_393_MOESM6_ESM.docx]

**Additional file 6: Table S6. Kaplan-Meier Complete and Overall Success Rates for IOP ≤ 21 and ≤ 17 mmHg**

|  |  | Kaplan-Meier Percent Success Rates (SE) | | | | |  |
| --- | --- | --- | --- | --- | --- | --- | --- |
|  |  | MMC | MMC |  | CM | CM | p-value |
|  |  | 1 year | 2 years |  | 1 year | 2 years | (log rank test) |
| IOP Cutoff 21 mm Hg | **Complete Success** | 51.8 (7.4) | 38.4 (7.6) |  | 67.2 (7.2) | 56.2 (7.9) | 0.112 |
|  | Exclude Combined | 51.9 (7.8) | 38.5 (7.8) |  | 62.8 (7.9) | 53.0 (8.5) | 0.243 |
|  | Stringent Definition of Hypotony | 54.0 (7.4) | 42.8 (7.7) |  | 67.2 (7.2) | 56.2 (7.9) | 0.230 |
|  |  |  |  |  |  |  |  |
|  | **Overall Success** | 65.1 (7.1) | 49.2 (7.8) |  | 86.0 (5.3) | 77.9 (6.6) | **0.007** |
|  | Exclude Combined | 64.1 (7.4) | 50.6 (8.0) |  | 84.1 (6.0) | 77.6 (7.1) | **0.014** |
|  | Stringent Definition of Hypotony | 67.3 (6.9) | 53.5 (7.8) |  | 86.0 (5.3) | 77.9 (6.6) | **0.020** |
|  |  |  |  |  |  |  |  |
|  |  |  |  |  |  |  |  |
| IOP Cutoff 17 mm Hg | **Complete Success** | 49.6 (7.4) | 35.9 (7.5) |  | 62.4 (7.4) | 51.4 (7.9) | 0.160 |
|  | Exclude Combined | 49.5 (7.8) | 35.9 (7.7) |  | 57.3 (8.1) | 47.6 (8.5) | 0.337 |
|  | Stringent Definition of Hypotony | 51.8 (7.4) | 40.3 (7.7) |  | 62.4 (7.4) | 51.4 (7.9) | 0.310 |
|  |  |  |  |  |  |  |  |
|  | **Overall Success** | 62.9 (7.1) | 44.1 (7.8) |  | 81.2 (6.0) | 65.4 (7.6) | **0.041** |
|  | Exclude Combined | 61.7 (7.5) | 45.3 (8.0) |  | 78.6 (6.7) | 63.3 (8.2) | 0.087 |
|  | Stringent Definition of Hypotony | 65.1 (7.1) | 48.3 (7.9) |  | 81.2 (6.0) | 65.4 (7.6) | **0.010** |
|  |  |  |  |  |  |  |  |
|  |  |  |  |  |  |  |  |
|  |  |  |  |  |  |  |  |
|  | Abbreviations: SE=standard error; MMC=mitomycin-C; CM=Collagen Matrix; IOP=intraocular pressure | | | | | | |
